# Prevalence and Correlates of Common Mental Disorders among Mothers of Young Children in Kilimanjaro Region of Tanzania

**DOI:** 10.1371/journal.pone.0069088

**Published:** 2013-07-03

**Authors:** Jacqueline G. Uriyo, Amina Abubakar, Mark Swai, Sia E. Msuya, Babill Stray-Pedersen

**Affiliations:** 1 Division of Women and Children, Oslo University Hospital, Rikshospitalet, Oslo, Norway; 2 Kilimanjaro Christian Medical University College, Moshi, Tanzania; 3 Department of Psychology, Tilburg University, Tilburg, The Netherlands; 4 KCMC Hospital, Moshi, Tanzania; 5 Department of Community Health, Kilimanjaro Christian Medical University College, Moshi, Tanzania; University of Pennsylvania, United States of America

## Abstract

**Background:**

Although poor maternal mental health is a major public health problem, with detrimental effects on the individual, her children and society, information on its correlates in low-income countries is sparse.

**Aims:**

This study investigates the prevalence of common mental disorders (CMD) among at-risk mothers, and explores its associations with sociodemographic factors.

**Methods:**

This population-based survey of mothers of children aged 0–36 months used the 14-item Shona Symptom Questionnaire (SSQ). Mothers whose response was “yes” to 8 or more items on the scale were defined as “at risk of CMD.”

**Results:**

Of the 1,922 mothers (15–48 years), 28.8% were at risk of CMD. Risk of CMD was associated with verbal abuse, physical abuse, a partner who did not help with the care of the child, being in a polygamous relationship, a partner with low levels of education, and a partner who smoked cigarettes. Cohabiting appeared to be protective.

**Conclusions:**

Taken together, our results indicate the significance of the quality of relations with one’s partner in shaping maternal mental health. The high proportion of mothers who are at risk of CMD emphasizes the importance of developing evidence-based mental health programmes as part of the care package aimed at improving maternal well-being in Tanzania and other similar settings.

## Introduction

Common mental disorders (CMD), including depression, anxiety and somatic symptoms, are reported to occur with more frequency among women than men, especially in low- and low-middle income countries (LLMICs) [1]. Poor maternal mental health has adverse social, economic and psychological consequences for the individuals, their children, their families and the community [2].

Reports from population-based studies show the prevalence of CMD among mothers as ranging from 10 to 56% [3–10]. The prevalence varies depending on the study design, the characteristics of the populations examined and the assessment instrument used.

Reported sociodemographic risk factors of CMD among mothers in LLMICs include being illiterate, having an unsupportive partner, physical violence, verbal abuse, alcohol use, polygamous marriage, having at least 3 children, HIV/AIDS and the death of a loved one [2,11–15].

However, there have been contrasting reports on the association between some demographic risk factors and CMD. While in some studies, mothers with CMD were more likely to be older [9,10,16] or younger [17,18], others found no association between age and CMD [5,7]. With regard to marital status, one study reported single, widowed, divorced or separated mothers to have a higher risk of CMD [4,19], contradicting results from other studies which showed that longer duration of marriage [16] and cohabiting [20] were positively associated with CMD. This indicates that in different settings, there is a need to determine local risk factors in order to provide the basis for intervention.

The Shona Symptom Questionnaire (SSQ) is a screening instrument for CMD which was developed and validated in Zimbabwe [21]. In contrast to diagnostic tools which give a diagnosis of the outcome, screening instruments identify those who are at a greater risk of the outcome. A comparison with 3 other instruments designed to detect psychiatric morbidity in community-based populations showed SSQ to be just as good as the others, with no consistent differences [22]. The brief nature of SSQ, and it’s sharing of a number of idioms and symptoms with those identified in some parts of Tanzania [23,24], makes it a practical screening tool to use in a population-based study.

This report is part of a community-based cross-sectional study that was conducted in Kilimanjaro, Tanzania. The main objective of the primary study was to document the development and the factors associated with poor development of children aged 0-36 months. The main aim of this report is to document the prevalence and factors associated with the risk of common mental disorders in mothers of children aged 0-36 months.

## Methods

### Ethics Statement

Prior to study commencement, the protocol was approved by the Kilimanjaro Christian Medical Centre Ethical Committee, the Tanzanian National Institute of Medical Research (NIMR) and the Regional Ethics Committee, South East Norway. Before joining the study, caregivers were informed about the study aims and procedures involved. Written consent was sought from the caregivers who wanted to join the study, and they were advised that they had the right to refuse to participate. In the case of illiterate caregivers, the right thumbprint was used as a signature.

### Design and Setting

From June 2010 to May 2011, the study team visited 50 villages/streets within 7 districts in Kilimanjaro region, Northern Tanzania. Kilimanjaro is projected to have a population of 1,702,207, with 451,911 females aged 15–49 years [25]. Approximately 80% of the inhabitants live in the rural areas. The total fertility rate is about 4.8 per woman, with the average household size ranging from 4–5 persons [25,26]. The majority of the inhabitants depend on subsistence farming. The predominant ethnic groups are the Chagga and Pare.

In principle, mental health is integrated into primary healthcare in Tanzania. At the community level, mental health-care is mainly provided by trained primary healthcare workers who are supervised by district mental health coordinators. However, this system faces a number of challenges including a shortage of skilled mental healthcare workers, insufficient funds for supervision, and a limited supply of essential medicines [27,28].

### Participants

Mothers of children aged 0-36 months were recruited for this study. There were no specific exclusion criteria.

#### Sampling Method

To obtain a representative sample of children aged 0-36 months for the original study, a multistage sampling design was used. First, a random cluster sampling method, with probability proportional to the size of the population, was used to select 50 clusters i.e. villages/streets within the 7 districts. This was done by listing the population of children aged 0-36 months for each village/street, with a column showing its cumulative population, to be used as a sampling frame. The village and street by single-age population data were obtained from the 2002 census (with 2009 projections), from the Kilimanjaro regional bureau of statistics office in April 2009. The sampling interval was then calculated: the total population of 0-36-month-olds was divided by the required number of clusters i.e. 166,808/50. A random number between 0 and 1 was generated from a computer, and the starting point for selection of the first cluster was determined by multiplying the random number with the sampling interval. The subsequent cluster was located by adding the sample interval to the previous number, until the 50^th^ cluster was selected. In each cluster, a compact segment sampling method was used to select 50 children aged 0-36 months. The selected clusters (i.e. villages/streets) were mapped into segments, each with approximately 50 children aged 0-36 months (from the 2009 projections of the 2002 census). All segments were assigned a number on pieces of paper, and one was randomly picked. Within the selected segment, a quota sampling method was used to determine the children to be included in the study. The study team visited all the households until 50 children whose parents consented and who fulfilled the gender and sex criteria were examined. If all the households in the segment had been surveyed and fewer than 50 children were available, a second segment was randomly selected. The members of the households in the selected segments were informed to be available on the day of the survey. The required sample size was 2,500 children, with approximately 100-200 children in each of the 12 specified age groups.

### Measures

#### Shona Symptom Questionnaire (SSQ)

The mothers’ mental health was screened using SSQ, a screening instrument designed to be used by lay interviewers for the measurement of non-psychotic psychological morbidities, including depressive, generalized anxiety and somatic disorders. The 14-item SSQ was developed using items from the WHO Self-Report Questionnaire, and 47 local idioms of distress of mental disorder that were identified by qualitative interviews of 110 patients with obvious psychiatric morbidities. The final list of 14 items of SSQ are made up of 7 items found in the SRQ (such as sadness, tearfulness and poor sleep), and 7 items locally identified and found to be relevant defining distress in the cultural context (such as perceptual hallucinations and ‘thinking too much’)

The validation of the SSQ was done using two validation criteria: (i) Revised Clinical Interview Schedule and (ii) Clinical diagnosis of mental problems by the care providers among patients attending primary health care clinics and traditional medical practitioners. Using a cut-off score of 8 or more as diagnosis of mental disorder, it had a reliable internal consistency (Cronbach’s alpha = 0.85), and the sensitivity and specificity of SSQ was 67% and 83% respectively [21]. . The SSQ can be used as either a self-completion questionnaire or an interviewer-administered tool. The participants scored ‘yes’ or ‘no’ if they had experienced symptoms in the previous week. Those who scored 8 or more are classified as ‘likely cases’ and the rest are classified as ‘noncases’ (Table 1). The SSQ was chosen as the main outcome measure in this study since it had been developed for use in a cultural context similar to the one in which we worked (viz. Zimbabwe).

**Table 1:** Shona Symptom Questionnaire (SSQ) Item Response Rate (n=1922).

**SSQ Items**	**Responded Yes**	
**In the course of the past week…**	n	%
**1. did you have times when you were thinking about many things or deeply?**	1095	57.0
**2. did you find yourself sometimes failing to concentrate**	690	35.9
**3. did you lose your temper or get annoyed over trivial matters**	1220	63.5
**4. did you have nightmares or bad dreams**	593	30.9
**5. did you sometimes see or hear things which others could not see or hear**	211	11.0
**6. was your stomach aching**	675	35.1
**7. were you frightened by trivial things**	731	38.0
**8. did you sometimes fail to sleep or lose sleep**	843	43.9
**9. were there moments when you felt life was so tough that you cried or wanted to cry**	885	46.0
**10. did you feel run down (tired)?**	899	46.8
**11. did you at times feel like committing suicide?**	190	9.9
**12. were you generally unhappy with things you were doing each day?**	457	23.8
**13. was your work lagging behind?**	643	33.5
**14. did you feel you had problems in deciding what to do?**	507	26.4

In the current study, the SSQ was translated into Swahili, back-translated, field-tested and revised accordingly. It was administered by the research assistants who consisted of two nurse midwives, a physiotherapist and a nutritionist, all of whom underwent training for two weeks. Mothers who scored above the cut-off of 8 were classified as ‘at risk’ and were referred for counselling and psychiatric assessment through the local health facility.

The factorial structure of the SSQ in this study was investigated using a principal component analysis. The scale has a strong first factor with an eigenvalue of 5.048, explaining 36.06% of the variance. All the items on the scale strongly loaded on this factor, with factor loadings ranging from 0.72 to 0.34. The unidimensionality of the scale was further confirmed by the high internal consistency (Cronbach’s alpha of 0.86).

#### Socio - demographic factors

The following information was obtained from the mothers using a structured interview guide: Maternal Information: age in years (less than 20, 20–29, 30–39, more than 39); marital status (single, married, cohabiting, widowed/divorced/separated); whether she was in a polygamous relationship; education level (less than 7 years, more than 7 years); employment status (housewife, farmer, small scale business, unskilled manual, professional/technical); residence (urban, rural); parity i.e. number of children ever borne (1-4, more than 4); if children were from the same partner; death of a child; whether she was verbally or physically abused by her partner in the past one month. The partner’s age in years(less than 30, 30–39, 40–49, more than 49); education level (less than 7 years, more than seven years); occupation (unemployed, farmer, small scale business, unskilled manual, professional/technical); alcohol and cigarette use were also recorded. The mothers who used alcohol both during pregnancy and during the study period were considered as ‘alcohol users’. Additionally, mothers were asked if their partner ever helped with taking care of the children e.g. changing clothes, bathing or feeding the child. Questions were asked about the most recent partner i.e. the father of the child in the study.

### Data Management and Statistical Analysis

Double data entry was done using EpiData version 3.1. Data checking, cleaning, recoding, labelling and analysis were done using PASW18 and Stata/IC 11.0. Frequency tables and univariate descriptive analysis were carried out to investigate the spread of scores. Bivariate analyses of those ‘at risk of CMD’ and possible associated factors were carried out using cross tabulations and χ^2^ tests. A p value was considered to be statistically significant when it was less than 0.05. The factors which were statistically significant in the univariate analysis were entered into the multivariate logistic regression model to determine significant factors associated with risk of CMD. The Odds Ratio (OR) and their 95% confidence interval (CI) were used to measure the strength of association between potential factors and risk of CMD.

## Results

### Sociodemographic characteristics of Mothers

Of the 2,774 households that were approached, 2,454 (88.5%) consented to participate in the study. Compared to participants, the non-participants were more likely to reside in urban areas (47.2 v 24.7%, *p* <0.001). Among the respondents, 2,354 (95.9%) were mothers, while the rest were caregivers. Mothers from one district (n=423, 18%) were excluded from this analysis because questions were added after collecting data in that area. The characteristics of these mothers differed significantly from the remaining mothers. In addition, 9 (0.4%) mothers were excluded because more than half of their information was missing.

Of the mothers (N = 1,922) included in this analysis, 1,456 (75.8%) were from rural areas. Their mean age and years of education were lower than their partners’ (28 + 7 v. 35 + 10 years) and (7.3 + 2.0 v. 8.0 + 3.2 years) respectively. The sociodemographic data are summarized in Tables 2 and 3.

**Table 2:** Sociodemographic factors associated with ‘Risk of CMD’ among 1922 mothers.

	**Total (n=1922)**	**Risk of CMD (n=553)**	**Unadjusted OR(95% CI)**		**Adjusted OR** ^1^ (**95%CI**)	
**Mother’s Characteristics**	**n (%)**	**n (%)**		***P*-value**		***P*-value**
**Age group in years**						
Less than 20	112 (5.8)	20 (17.9)	1		1	
20-29	1016 (53.0)	275 (27.1)	1.71 (1.03-2.82)	0.037	1.48 (0.85-2.58)	
30-39	660 (34.3)	204 (30.9)	2.06 (1.23-3.43)	0.006	1.25 (0.67-2.32)	
40+	118 (6.1)	48 (40.7)	3.15 (1.72-5.79)	<0.001	1.60 (0.74-3.45)	
Don’t know	16 (0.8)	6 (37.5)	2.76 (0.90-8.47)	0.076	1.61 (0.44–5.90)	
**Years of education**						
More than 7	311 (16.2)	61 (19.6)	1		1	
7 or less	1611 (83.8)	492 (30.5)	1.80 (1.34-2.43)	<0.001	0.99 (0.69-1.41)	
**Marital Status**						
Single	236 (12.3)	82 (27.4)	1		1	
Married	1063 (55.3)	394 (30.1)	1.11 (0.82-1.52)	0.487	0.99 (0.66-1.49)	
Cohabiting	554 (28.8)	128 (19.5)	0.60 (0.42-0.85)	0.004	0.51 (0.33-0.79)	0.002
Widowed/Divorced/Separated	69 (3.6)	36 (44.4)	1.93 (1.12-3.35)	0.019	1.07 (0.57–2.00)	
**Polygamous relationship**						
No	1705 (88.7)	467 (27.4)	1		1	
Yes	217 (11.3)	86 (39.6)	1.74 (1.30–2.33)	<0.001	1.41 (1.00-1.98)	0.048
**Occupation (n=2300)**						
Professional/Technical	106 (5.6)	19 (17.9)	1		1	
Unskilled manual	143 (7.4)	58 (40.6)	3.12 (1.72-5.68)	<0.001	1.69 (0.87-3.29)	
Small scale business	513 (26.7)	130 (25.3)	1.55 (0.91-2.65)	0.106	1.01 (0.57-1.80)	
Farmer	810 (42.1)	266 (32.8)	2.24 (1.33-3.75)	0.002	1.51 (0.85-2.69)	
Housewife	350 (18.2)	80 (22.9)	1.36 (0.79-2.36)	0.282	1.00 (0.55-1.82)	
**More than 4 children**						
No	1582 (82.3)	428 (27.1)	1		1	
Yes	340 (17.7)	125 (36.8)	1.57 (1.22–2.01)	<0.001	0.95 (0.67-1.33)	
**Children of the same partner**						
Yes	1604 (83.5)	433 (27.0)	1		1	
No	318 (16.5)	120 (37.7)	1.64 (1.27–2.11)	<0.001	1.29 (0.97-1.73)	
**Drinks alcohol**						
No	1161 (60.4)	292 (25.2)	1		1	
Yes	761 (39.6)	261 (34.3)	1.55 (1.27–1.90)	<0.001	1.21 (0.94-1.55)	
**Reported HIV status**						
Negative/Not known	1849 (96.2)	522 (28.2)	1		1	
Positive	73 (3.8)	31 (42.5)	1.88 (1.17–3.02)	0.008	1.40 (0.83–2.37)	

^1^ Adjusted for all factors in Tables 2 and 3

**Table 3:** Sociodemographic factors associated with ‘Risk of CMD’ among 1922 mothers.

	**Total (n = 1922)**	**Risk of CMD (n=553)**	**Unadjusted OR (95% CI)**		**Adjusted OR** ^1^ (**95%CI**)	
**Partner’s Characteristics**	**n (%)**	**n (%)**		***P*-value**		***P*-value**
**Age group in years**						
Less than 30	526 (27.4)	128 (24.3)	1		1	
30-39	776 (40.4)	234 (30.2)	1.34 (1.04-1.73)	0.022	1.11 (0.82-1.50)	
40-49	349 (18.2)	107 (30.7)	1.37 (1.02-1.86)	0.039	0.94 (0.63-1.42)	
More than 49	81 (4.2)	36 (44.4)	2.49 (1.54-4.03)	<0.001	1.65 (0.89–3.01)	
Not known	190 (10.0)	48 (25.3)	1.05 (0.72-1.54)	0.799	0.30 (0.16–0.58)	<0.001
**Years of education**						
More than 7	448 (23.3)	87 (19.4)	1		1	
7 or less	1355 (70.5)	420 (31.0)	1.86 (1.44–2.42)	<0.001	1.54 (1.11-2.14)	0.009
Not known	119 (6.2)	46 (38.7)	2.61 (1.69–4.05)	<0.001	3.29 (1.43–7.55)	0.005
**Occupation**						
Professional	327 (17.0)	75 (22.9)	1		1	
Unskilled manual	470 (24.5)	160 (34.0)	1.73 (1.26-2.39)	0.001	1.12 (0.77-1.63)	
Small scale business	524 (27.3)	140 (26.7)	1.22 (0.89-1.69)	0.217	0.90 (0.62-1.31)	
Farmer	437 (22.7)	126 (28.8)	1.36 (0.98-1.89)	0.067	0.75 (0.50-1.13)	
Unemployed	18 (0.9)	4 (22.2)	0.96 (0.31–3.00)	0.944	0.43 (0.12-1.55)	
Not known	146 (7.6)	98 (32.9)	1.65 (1.07–2.53)	0.023	1.05 (0.54–2.05)	
**Drinks alcohol**						
No	710 (37.0)	159 (22.4)	1		1	
Yes	1119 (58.2)	359 (32.1)	1.64 (1.32-2.03)	<0.001	1.03 (0.79-1.35)	
Not known	93 (4.8)	35 (37.6)	2.09 (1.33–3.30)	0.001	0.70 (0.21–2.35)	
**Smokes cigarettes**						
No	1334 (69.4)	333 (25.0)	1		1	
Yes	495 (25.8)	183 (37.0)	1.76 (1.41-2.20)	<0.001	1.37 (1.06-1.77)	0.016
Not known	93 (4.8)	37 (39.8)	2.00 (1.29–3.06)	0.002	2.81 (0.95–8.33)	
**Verbally abused by partner**						
No	1577 (82.0)	365 (23.1)	1		1	
Yes	345 (18.0)	188 (54.5)	3.98 (3.12-5.06)	<0.001	2.90 (2.12–3.97)	<0.001
**Physically abused by partner**						
No	1766 (91.9)	459 (26.0)	1		1	
Yes	156 (8.1)	94 (60.3)	4.32 (3.08-6.05)	<0.001	1.69 (1.09-2.61)	0.019
**Partner assists with caring for child(feeding, clothing)**						
Yes	1546 (80.4)	398 (25.7)	1		1	
No	376 (19.6)	155 (41.2)	2.02 (1.60–2.56)	<0.001	2.03 (1.51–2.75)	<0.001

^1^ Adjusted for all factors in Tables 2 and 3

### Risk of Common Mental Disorders

Overall, 553 (28.8%) mothers scored 8 or more on the scale, and hence were considered to be at risk of CMD. The least-reported symptoms were suicidal ideation and hallucination. Table 1 shows the SSQ item response rate. Although the difference was not statistically significant, the proportion of those at risk in the rural areas was higher than in the urban areas (29.7 and 25.8%, χ^2^ (DF, 1; N, 1992) 2.74, *p* = 0.098).

### Factors Associated with Risk of Common Mental disorders

An initial bivariate analysis (Tables 2 and 3) showed that the factors significantly associated with risk of CMD were: mother’s age, years of education, marital status, occupation, parity, having children with different partners, alcohol use, physical abuse, verbal abuse, polygamous relationship and disclosing their HIV-positive status to the interviewer. Partner’s age, occupation, years of education, partner who did not help with care of the child, alcohol and cigarette use were also significantly associated with maternal risk of CMD. Death of a child was not associated with risk of CMD (OR = 1.01, 95% CI 0.71-1.43).

After controlling for confounding factors (Tables 2 and 3), verbal abuse, physical abuse, partner who did not help with care of the child, being in a polygamous relationship, partner with low or no education and cigarette smoking remained independently associated with risk of CMD. Cohabiting was negatively associated with risk of CMD. Furthermore, mothers who did not know their partner’s characteristics were also more likely to be at risk of CMD.

## Discussion

The results of this study suggest that more than 1 in 4 mothers are at risk of CMD. The prevalence of mothers at risk of CMD reported here is consistent with reports from other LLMICs [7,9–11,16,29]. These findings further emphasize the need to prioritize measures aimed at alleviating CMD among mothers in the LLMIC.

Consistent with several other reports, in this study intimate partner verbal and physical abuse also independently predicted risk of CMD [8,9,11,30,31]. Of the two, verbal abuse was more frequent (18 v. 8%) and it was the strongest predictor of risk of CMD. Contrary to our findings, the 2010 Tanzania Demographic and Health Survey (TDHS) reported physical violence to be more frequent than emotional/verbal violence (25.5 v. 13.6%) among women aged 15-49 years from Kilimanjaro region [26]. Due to the sensitive nature of the questions on physical abuse in this culture, it is possible that some mothers in this study did not respond truthfully. In addition, the fact that fewer women participated in the 2010 TDHS survey could explain the contradictory differences observed. Regardless of the slight difference in the pattern of results, these findings place CMD within the context of marital relations, and point to the potential saliency of having a family-centred approach to addressing maternal mental health problems.

Evidence suggests that alcohol use by either or both partners increases the severity of intimate partner violence [26,32–34]. However, in this study, neither maternal nor partner alcohol use were independent predictors of risk of CMD.

Being married as opposed to being single was independently associated with risk of CMD in Indian women [35]. This is comparable to the trend seen in this study. Compared with single or married mothers, mothers in this study who were cohabiting were less at risk of CMD. However, one study found the risk of depression to be 20 times higher in cohabiting postpartum mothers [36]. The 2010 TDHS reported that never-married women (6%) were less likely to experience physical abuse during pregnancy when compared with married (8%) women [26]. Does abuse mediate the effect of marital status on risk of CMD?

Education provides the means to cope with difficulties and to extricate oneself from poverty. Although the association between risk of CMD and the mother’s low level of education was not consistent in this study, the partner’s low level of education predicted CMD in the mother. This is consistent with a study which showed mothers with uneducated husbands to have a higher risk of persistent depression even beyond the postpartum period [37]. In results not reported here, partner level of education was correlated to reported violence, with partners who are less educated being more likely to commit acts of violence. This implies that violence may be the mediating factor between partners’ educational level and CMD. Future work aimed at teasing out this complex relationship is warranted.

Mothers in this study with partners who helped with child care were less likely to be at risk of CMD. In one study, mothers who got daily support with child care from at least one family member were less likely to have depression [7]. These results, together with earlier observations, point to the potential importance of the quality of marital relations and the family environment in determining maternal mental health.

HIV-infected persons with symptoms of depression were reported to have an increased risk of disease progression and poorer quality of life [15,38]. The mothers who disclosed their HIV-positive status to the interviewers in this study were more at risk of CMD than the others. Fear of the disease and its consequences, self-stigma, and the discrimination to which people living with HIV infection in Tanzania are still subjected, could contribute to the high proportion of risk of CMD that is seen [39,40].

The current study used the SSQ to evaluate the existence of the CMD in a large population-based study. The use of SSQ is a potential limitation since it is too brief to provide any diagnosis; however, since our aim was to screen and not diagnose, we do not find the brevity of SSQ to compromise the validity of our findings. Additionally, in the present state, the SSQ is uniquely placed to perform well in a rural Tanzanian setting, since it was developed using emic and etic approaches to suit the African cultural setting when discussing mental health issues and its simplicity allows for its use by staff that may be of relatively limited educational background.

Among the limitations of this study is the refusal rate of working mothers in the urban areas to participate in the study. This could have influenced the results, as there might have been fewer mothers with higher levels of education. The verbal diagnosis of HIV used in this study could have resulted in an underestimation of the number of HIV-infected mothers. Furthermore, this was a cross-sectional study, and hence it is difficult to comment on the direction of association between partner abuse and risk of CMD. Earlier reports on the mental health effects of intimate partner violence suggest that CMD results from intimate partner violence [34,41,42]. On the other hand, there is paucity of evidence suggesting that CMD could precipitate acts of violence from the partner. In addition, the definition of mothers ‘at risk of CMD’ used a SSQ cut-off value that has not been validated in this population. Lastly, the primary question in this study required that we used a sampling frame that was not truly random. The multistage quota sampling may have introduced biases resulting from this approach, however given that the pattern of our results closely mirror what has been observed elsewhere, we doubt if this would be a threat to the validity of our findings and conclusions.

In conclusion, the risk of CMD among mothers in this population is high. Results from this study emphasize the association between partner abuse, low levels of education, and lack of partner support in child care with risk of CMD. A culture which encourages partners to be involved in child-caring activities might also contribute to decreasing the risk of maternal CMD. A longitudinal study to establish the local risk factors for CMD would provide the basis for creating a screening tool for community use. In addition, primary healthcare providers should be provided with skills and tools to detect and manage CMD in the community.

The risk of CMD among mothers of young children in our study area is a source of concern, as it is likely to lead to adverse effects on child and maternal health. There is a need to invest resources with the aim of fully understanding risk factors for CMD and developing evidence-based mental health services in order to enhance maternal mental health. 
